# C/EBPβ Mediates Osteoclast Recruitment by Regulating Endothelial Progenitor Cell Expression of SDF-1α

**DOI:** 10.1371/journal.pone.0091217

**Published:** 2014-03-11

**Authors:** Sheng-Long Fu, Hao Pang, Jian-Zhong Xu, Xue-Hui Wu

**Affiliations:** National & Regional Engineering Laboratory of Tissue Engineering, Department of Orthopaedics, Southwest Hospital, Third Military Medical University, Chongqing, China; Virginia Commonwealth University, United States of America

## Abstract

Integration of tissue-engineered bone grafts with the host bone is vital for the healing of critical-size bone defects. An important aspect of this process is bone resorption, which must be carried out by osteoclasts derived from the host. However, the mechanism underlying recruitment of host osteoclast precursors to graft sites remains unclear. Endothelial progenitor cells (EPCs) mobilize from the bone marrow into the circulation and home to sites of angiogenesis such as tissue remodeling. Since EPCs express SDF-1, and C/EBPβ is known to regulate SDF-1α expression, we hypothesized that EPCs may recruit CXCR4-expressing host osteoclast precursors to the repair area and that this recruitment may be mediated through C/EBPβ signaling. Using an inflammatory EPC model we showed that EPCs upregulate protein levels of both SDF-1α and C/EBPβ. A luciferase assay confirmed that C/EBPβ acts on the SDF-1α promoter in these cells, and that binding is increased under conditions of inflammation, while silencing of C/EBPβ reduces expression of SDF-1 α and C/EBPβ. Using RAW264.7 cells as a model of osteoclastic monocyte precursors, we investigated their responses to migratory factors in EPC conditioned medium. We showed that RAW264.7 cells migrate towards conditioned medium from EPCs treated with IL-1β, an effect which could be abolished by silencing C/EBPβ in EPCs, and was almost completely blocked by silencing CXCR4 in RAW264.7 cells. These findings show that EPCs respond to inflammatory stimuli by signaling to osteoclast precursors via SDF-1, and that C/EBPβ mediates this response.

## Introduction

Critical-size bone defects cannot heal naturally and must be replaced by tissue engineered bone (TEB) [1]. A variety of materials have been used to produce grafts, but any scaffold materials used must be biodegradable and allow integration with native bone [2]. Both vascularization and new bone formation are important in this process, together with later bone remodeling [1]. Early activation of osteoclasts is beneficial for resorption of necrotic bone at the fracture end, and crucial for bone reconstruction and shaping in the later stages of integration and healing [2].

Osteoclasts are the bone-resorbing cells which, together with bone-forming osteoblasts, are responsible for coordinated bone remodeling. They are derived from hematopoietic precursors of the monocyte-macrophage lineage residing in the bone marrow [3]. Osteoclast development, growth, survival and activity are regulated by cells of the osteoblastic lineage through their expression of receptor-activator of NFκB ligand (RANKL) and its soluble decoy receptor osteoprotegerin (OPG). RANKL binds to its receptor, RANK on the surface of osteoclast precursors, and is inhibited by OPG [4]. RANKL is both necessary and sufficient for osteoclast differentiation and activity [5] as well as survival [6], although macrophage-colony stimulating factor (M-CSF) is also important in osteoclast and pre-osteoclast survival, proliferation and maturation [7].

Although regulation of osteoclast differentiation has now been elucidated, control of the migration of osteoclast precursors from the bone marrow and peripheral circulation and homing to sites of bone resorption remains poorly understood. Chemokines are small chemotactic cytokines that are known to regulate inflammatory processes and cell trafficking. The CXC chemokine, stromal cell-derived factor (SDF)-1/CXCL12, and its G-protein-coupled receptor, CXCR4, are involved in cellular chemotaxis, angiogenesis, and cell proliferation [8], [9], and are involved in controlling development of the hematopoietic and vascular systems as well as playing a role in cancer metastasis [10].

Endothelial progenitor cells (EPCs) come from the bone marrow, and give rise to cells of the endothelial lineage. They mobilize from the bone marrow into the circulation and home to sites of angiogenesis, for example tissue remodeling and regeneration after vascular injury [11]. They also play a key role in neovasculogenesis in response to the hypoxic and thus inflammatory microenvironment which develops within sites such as rapidly-growing tumors and ischemic tissues [12], [13]. EPCs can promote the early vascularization of tissue engineered bone, and further promote osteogenesis, thus accelerating bone healing [14].

When TEB is used to repair a bone defect, large numbers of host osteoclast precursors are recruited to the site of the defect and involved in bone resorption. The underlying mechanism involves inflammatory factors (such as IL-6, IL-1, TNF-alpha) produced as a result of the local trauma of the surgery when TEB is implanted into the bone defect site [1]. The foreign body response is also induced by introduction of any implants including biomaterials. The foreign body reaction consists of several stages, beginning with inflammatory cell infiltration and blood–material interactions, progressing through acute and chronic inflammatory stages and the formation of granulation tissue and finally culminating in fibrous capsule formation. Monocytes and macrophages are involved in each stage and eventually form foreign body giant cells. During all of these stages the cells of the monocyte/macrophage lineage produce and secrete a range of cytokines involved in inflammation [15]. These released inflammatory factors stimulate seed cells including EPCs, which can produce cytokines and chemokines in the local environment [16]. The cytokines and chemokines stimulate migration and infiltration of host monocytes and recruit osteoclast precursors and associated cells such as mononuclear macrophages.

EPCs are known to express SDF-1 [17] and its receptor CXCR4 [18]. Since C/EBPβ is known to regulate SDF-1α expression, we hypothesized that EPCs recruit host osteoclast precursors, which express high levels of CXCR4 [19], [20], to the repair area and that this recruitment is mediated through C/EBPβ signaling which is thus involved in regulating the balance between bone formation and resorption. C/EBPβ is well known to be a key player in the regulation of osteogenesis, expressed in increasing amounts during osteoblast differentiation from mesenchymal stem cells and interacting with the transcription factor RUNX2 during skeletal growth to activate the osteocalcin promoter [21], [22].

## Materials and Methods

### Ethics statement

All animal experiments were approved by the medical ethics committee of Chongqing Southwest Hospital and were performed in accordance with established International Guiding Principles for Animal Research.

### Preparation of EPCs and cell culture

Male C57BL/6 mice, 6–8 weeks old, (purchased from the Animal House Center of Fudan University School of Medicine, Shanghai, China) were used in this study. Animals were euthanized by i.v. anesthesia with ketamine (25 mg/kg) and xylazine (2 mg/kg). The femora and tibiae were removed under sterile conditions and marrow was flushed from the bones using sterile serum-free medium. Bone marrow-derived mononuclear cells were then separated by density gradient centrifugation with Ficoll-Isopaque Plus (Histopaque-1077, Sigma-Aldrich, St Louis, MO). Isolated mononuclear cells were plated at a density of 1×10^6^ cells/cm^2^ on fibronectin-coated dishes (BD Biosciences, San Jose, CA) and cultured in EGM-2 endothelial medium (Lonza, Walkersville, MD), supplemented with 10% FBS (Thermo Scientific Hyclone, Logan, UT).

### Cell staining

After four days of culture, EPCs, recognized as attaching spindle-shaped cells, were characterized by fluorescent staining and flow cytometry. EPCs were identified by their ability to take up 1,1′-dioctadecyl-3,3,3′,3′-tetramethylindocarbocyanine (DiI)-labeled acetylated low density lipoprotein (acLDL, Biomedical Technologies, Stoughton, MA), and bind to FITC-labeled lectin (Ulex europaeus agglutinin (UEA)-1, Sigma) as described previously [23]. Briefly, cells were incubated with 2.5 µg/mL acLDL at 37°C for 3 hours, then fixed with 2% paraformaldehyde for 10 min. After washes, the cells were counterstained with 10 µg/mL FITC-labeled UEA-1 for 1 h at 37°C. Stained cells were viewed with an inverted fluorescent microscope (Nikon) and those exhibiting double-positive fluorescence were identified as EPCs.

### Flow cytometric analysis

In parallel, flow cytometric analysis was used to assess EPC surface markers. Adherent cells were detached by scraping with accutase (Sigma-Aldrich), then PE-conjugated anti-CD133 and anti-KDR, FITC-conjugated anti-CD34 and anti-CD31 antibodies (Jackson IRL, Baltimore, MD) were added and the suspension was incubated for 45 min at room temperature. Isotype-matched antibodies served as controls. After washing, cells were analyzed on an FC500 flow cytometer (Becton Dickinson, Fullerton, CA), and data were analyzed with CellQuest software (Becton Dickinson). The data were collected from 20,000 cells for each sample.

### Luciferase assay

A 1965 bp fragment of SDF-1 alpha promoter was amplified by PCR from mouse genomic DNA using the following primers: forward, 5′-TGGGGTACCAGGAGACCTGCAGACTT-3′, reverse, 5′- ACCAAGCTTGAGCAAAGAGACCAAACA-3′. The PCR product was cloned into the pGL3 basic luciferase reporter plasmid (Promega, Madison, WI) to generate the plasmid pGL3-SDF-1α promoter. To generate the mutant reporter vector, the SDF-1α promoter missing the C/EBP beta link site was amplified with the following primers: forward, 5′-CCCGGGTACCCTGAGAAGGTCAAAGGGAG-3′, reverse, 5′-ACCAAGCTTGAGCAAAGAGACCAAACA-3′. DNA fragments were cloned into the pGL3 basic luciferase reporter plasmid to generate the plasmid pGL3-SDF-1α mut. Restriction sites (*Kpn*I and *Hind*III) were added to the 5′ ends of the primers (*Kpn*I: GGTACC, *Hind*III: AAGCTT). pGL3-Basic Vector and pRL-TK Vector were purchased from Promega. pCMV-C/EBPβ and negative control pCMV DNA were purchased from Invitrogen.

The plasmids were co-transfected into normal EPCs in 24-well plates together with the pRL-TK plasmid (Promega) using Lipofectamine reagent (Invitrogen, Carlsbad, CA) and pCMV-C/EBPβ or negative control pCMV DNA (Ambion, Austin, TX). After 48 h, cells were washed in phosphate-buffered saline and processed according to the instructions for luciferase detection using dual specificity reporter gene kits (Promega). Relative luciferase activity was measured using a luminometer (Wallac Oy, Turku, Finland).

### Chromatin immunoprecipitation (ChIP) assay

ChIP analysis was performed using a ChIP assay kit (Upstate Biotechnology, Lake Placid, NY). Briefly, EPCs after 24 h treatment with 10 ng/mL IL-1β or control vehicle (DMSO) were fixed by adding formaldehyde to a final concentration of 1% at 37°C for 10 min and then washed with ice-cold PBS twice, collected by centrifugation (5 min at 2000 × *g*) and resuspended in 1 mL of SDS lysis buffer containing 1 × protease inhibitor cocktail (Roche, Rotkreuz, Switzerland). Cell lysate was sonicated (5 times with 10-s pulses and 1-min breaks) on ice with a 60 Sonic Dismembrator (Fisher Scientific), followed by centrifugation at 4°C for 10 min. Immunoprecipitations were carried out according to the manufacturer’s protocol. For the SDF-1α promoter, soluble chromatin was incubated with 2 µg of anti-C/EBPβ (Abcam, Cambridge, UK). Negative controls were incubated with rabbit immunoglobulin (Ig) G (Santa Cruz Biotechnology, Santa Cruz CA). PCR reactions were carried out using *ExTaq* Hot Start DNA polymerase (Takara Shuzo, Otsu, Japan).

### Lentivirus vector structure and transfection

Lentiviral vectors containing small hairpin RNA (shRNA) were constructed [24] using the sequences: C/EBP–β: GAAGAAACGTCTATGTGTA; CXCR4: GGAGGGGATCAGTATATAC; shRNA control: TTCTCCGAACGTGTCACGT. The shRNA oligonucleotides were annealed and cloned into the pLVTHM transfer plasmid (Invitrogen) at the *Mlu*I/ *Cla*I site. Recombinant lentivirus was produced by transient transfection of 293T cells using Lipofectamine 2000 (Invitrogen). Medium containing infectious lentiviruses was harvested at 48 h post-transfection and filtered through 0.45-µm-pore cellulose acetate filters.

EPCs were incubated with medium containing infectious lentiviruses carrying Lenti-shC/EBP–β or Lenti-shRNA plasmid, and RAW264.7 cells (purchased from Cell Bank of China Science Academy, Shanghai, China) were incubated with medium containing infectious lentiviruses carrying the Lenti-shCXCR4–β or Lenti-shRNA plasmid.

### Western blotting

Cells were lysed in buffer and total protein content was determined using the BCA protein Assay Kit (Beyotime). Protein expression was measured by western blot as previously described [18]. Briefly, proteins were separated by 10% sodium-dodecylsulphate-polyacrylamide gel electrophoresis (SDS-PAGE) and then transferred onto nitrocellulose membranes (Millipore, Bedford, MA). After blocking in Tris buffered saline containing 0.1% Tween-20 (TBS-T) with 5% nonfat dry milk for 30 min, membranes were washed 4 times in TBS-T and incubated with primary antibodies overnight at 4°C. Primary antibodies were all obtained from Abcam (Cambridge, UK) and used at the following dilutions: anti-SDF-1α (1/10,000), anti-CEBPβ (1/100), anti-β actin (1/500) and anti-CXCR4 antibody (1/2,000). After washing they were then incubated with the respective peroxidase-conjugated goat anti-rabbit IgG (1/2,000, Abcam) for 1 h at room temperature. The membranes were washed again three times, and then detected using an enhanced chemiluminescence reagent kit (Amersham Life Science, Cleveland, OH). The densitometric values were determined using a gel image analysis system (Bio-Rad, Hercules, CA) normalized to β-actin.

### Real-time quantitative RT-PCR

Total RNA was isolated from transfected and control cells using RNeasy mini kits (Qiagen, Inc., Valencia, CA), and treated with RNase-free DNAse to remove contaminating genomic DNA (Qiagen). One microgram of total RNA was used for first-strand DNA synthesis with Im-Prom RT and a dT primer. QRT-PCR was performed in an ABI Prism 7000 using SYBR Green master mix (Applied Biosystems) according to the manufacturer’s instructions [25].

Amplification used the following primers:

SDF-1 alpha, F: 5′-TGCATCAGTGACGGTAAACCA-3′,

R: 5′-TCAGCCGTGCAACAATCTGA-3′

C/EBPβ, F: 5′-GCCAACTTCTACTACGAGCCC-3′,

R: 5′-TTGTACTCGTCGCTCAGCTTG-3′

CXCR4, F: 5′-CCGTGTTCCTACCCCCAATG-3′,

R: 5′-GTCCACCACCCTGTTGCTGTA-3′

GAPDH, F: 5′-GTGCTATCCCTGTACGCCTC-3′,

R: 5′-GGCCATCTCTTGCTCGAAGT-3′

Amplification was monitored in real time until a significant level of fluorescence (C_t_) was reached. Products were quantified using the 2^−▵▵Ct^ method and expression is shown relative to GAPDH.

### ELISA

Untreated EPCs or EPCs transfected with Lenti-shC/EBPβ or Lenti-shRNA for 12 h were treated with 10 ng/mL IL-1β or control vehicle (DMSO) for 24 h. At the end of the incubation period, conditioned medium was collected, centrifuged at 200 × *g* and stored at -80°C until used to determine SDF-1α secretion by ELISA [26]. A specific SDF-1α ELISA kit (R&D Systems, Minneapolis, MN) was used, with a minimum detectable concentration of 18 pg/mL.

### Chemotaxis assay of RAW264.7 cells

Chemotaxis of RAW264.7 cells was assessed using a modification of a previously-described method [10]. Briefly, 200 µL EGM-2 endothelial medium or the supernatants of EPC cultures supplemented with 10 ng/mL IL-1β for 24 h (untreated EPCs, Lenti-shC/EBPβ, or Lenti-shRNA-EPCs) were placed in the lower chambers of a 24-well plate (Corning, New York, NY). These were separated from the upper chambers by an 8 µm pore size polycarbonate membrane containing 4×10^5^ untransfected RAW264.7 cells maintained in plain EGM-2 medium or supplemented with IL-1β (10 ng/mL) or SDF-1α (Bio Basic Inc., Ontario, Canada; 100 ng/mL), or Lenti-shCXCR4-RAW264.7 cells treated with IL-1β. Plates were incubated at 37°C in 5% CO_2_ in air for 8 h, after which the membranes were removed and non-migrating RAW264.7 cells were scraped from the upper surface. Membranes were then fixed in 4% paraformaldehyde and stained with hematoxylin, and numbers of migrated cells on the lower surface of the membrane were counted in 10 random high-power fields under a light microscope (Zeiss, Oberkochen, Germany); results are expressed as the chemotactic index (CI).

### Statistical analysis

All data were expressed as means ± SD from triplicate experiments performed in a parallel manner unless otherwise indicated. For statistical analysis of the data, group means were compared by one-way ANOVA, and Bonferroni’s test was used to identify differences between groups. Statistical differences were considered significant at the ^*^ *P*<0.05 or ^**^ *P*<0.01 level. All the data shown were obtained from at least three independent experiments.

## Results

### Properties of EPCs

**Figure 1:**
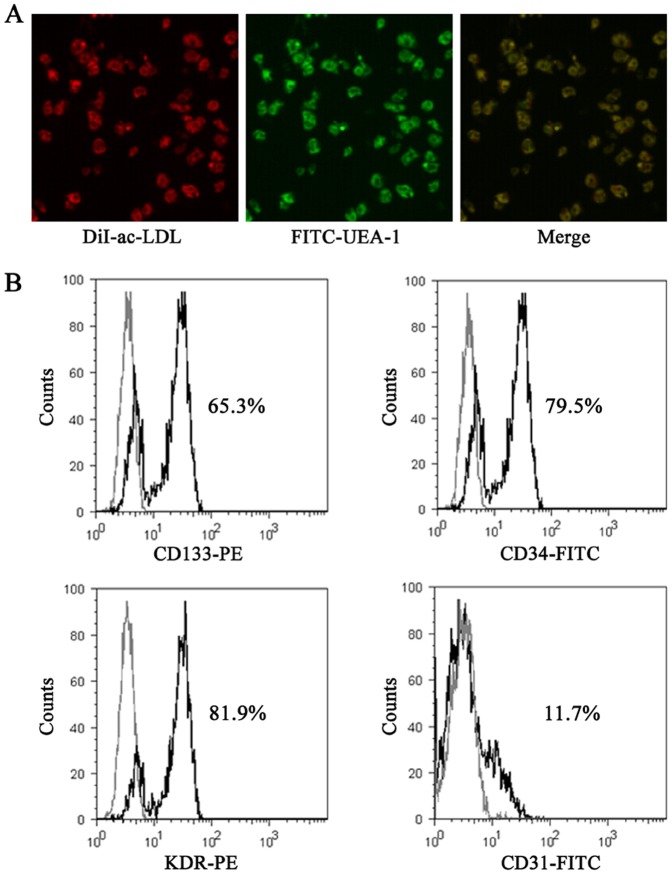
Confirmation of the EPC phenotype. **A**, Co-staining with DiI-acLDL and FITC-UEA confirmed that the cell population expressed both markers (magnification 100×). **B**, Cells were labeled with CD133/CD34/KDR/CD31 (black lines) and analyzed by flow cytometry; labeled cells are shown compared with controls stained with isotype-matched antibodies (gray lines). The results showed that the majority of the isolated cells were positive for the EPC surface markers CD133, CD34 and KDR, but negative for CD31

The phenotype of the EPCs was confirmed by dual-label staining with DiI-acLDL and FITC-UEA, which has been previously shown to positively identify a large proportion of EPCs [27] (Fig. 1A). Flow cytometric analysis showed that the isolated cells were positive for the EPC surface markers CD133, CD34 and KDR, but negative for CD31 (Fig. 1B).

### EPCs respond to IL-1β by upregulating protein levels of both SDF-1α and C/EBPβ

**Figure 2:**
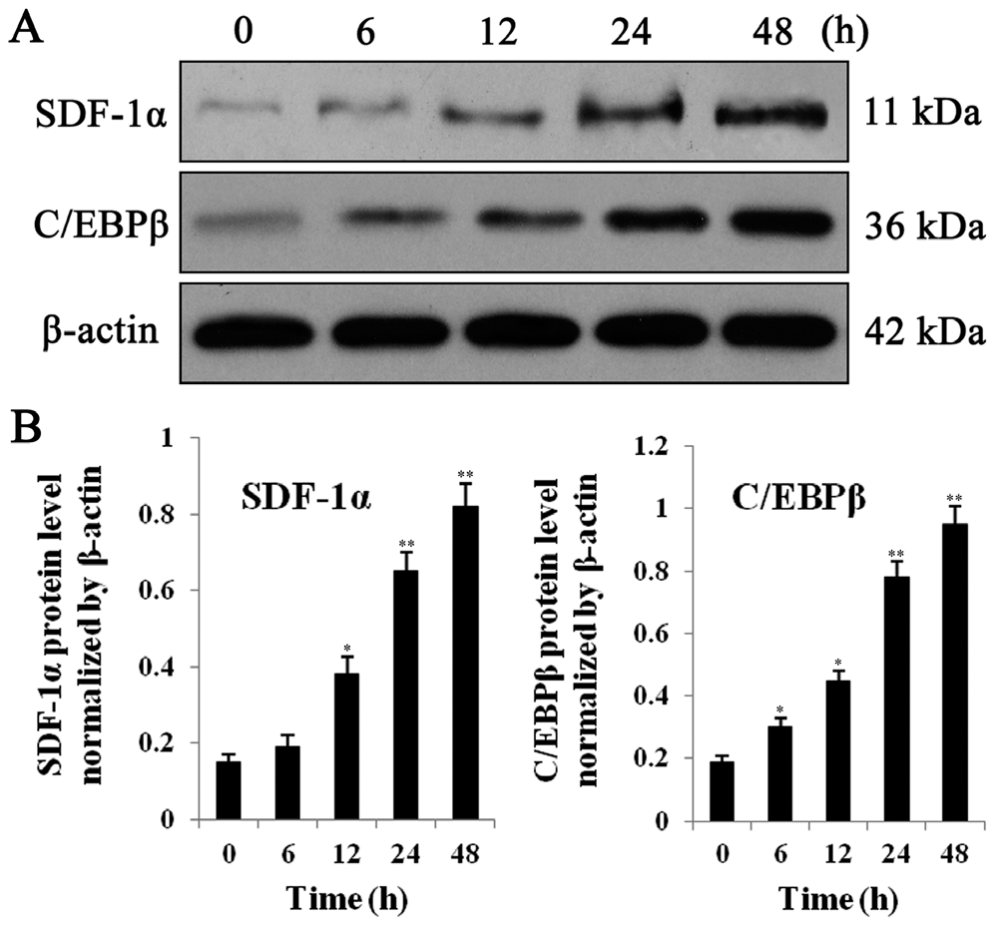
Protein expression of SDF1α and C/EBP-β. **A,** EPCs were incubated for 48 h in medium with human recombinant IL-1β (Endogen, Cambridge, MA) at a concentration of 10 ng/mL, then protein levels of SDF1α and C/EBP-β were detected at 0, 6, 12, 24, 48 h by western blot analysis. β-actin served as loading control. **B,** Quantitative analysis of SDF1α and C/EBP-β protein levels normalized to β-actin. All values are the means ± SD of three replicates, ^*^ *P*<0.05, ^**^ *P*<0.01 vs. 0 h.

Expression of both C/EBPβ and SDF-1α has been shown to be stimulated under inflammatory conditions characterized by high levels of IL-1β, and previous studies have used IL-1β to reproduce these effects [28], [29]. To test our hypothesis that C/EBPβ regulates expression of SDF-1α, we therefore investigated their induction by IL-1β in EPCs. To investigate whether the EPCs responded to the inflammatory cytokine IL-1β by regulating SDF-1α and C/EBPβ, cells were incubated with recombinant IL-1β (Endogen, Cambridge, MA) at 10 ng/mL for 48 h. At a range of time points as shown (Fig. 2A), cells were lysed and proteins detected by western blotting; expression was normalized to β-actin. Quantification of the blot results showed that IL-1β significantly increased levels of both SDF-1α and C/EBPβ proteins in a time-dependent manner (Fig. 2B).

### C/EBPβ acts on the SDF1α promoter

**Figure 3:**
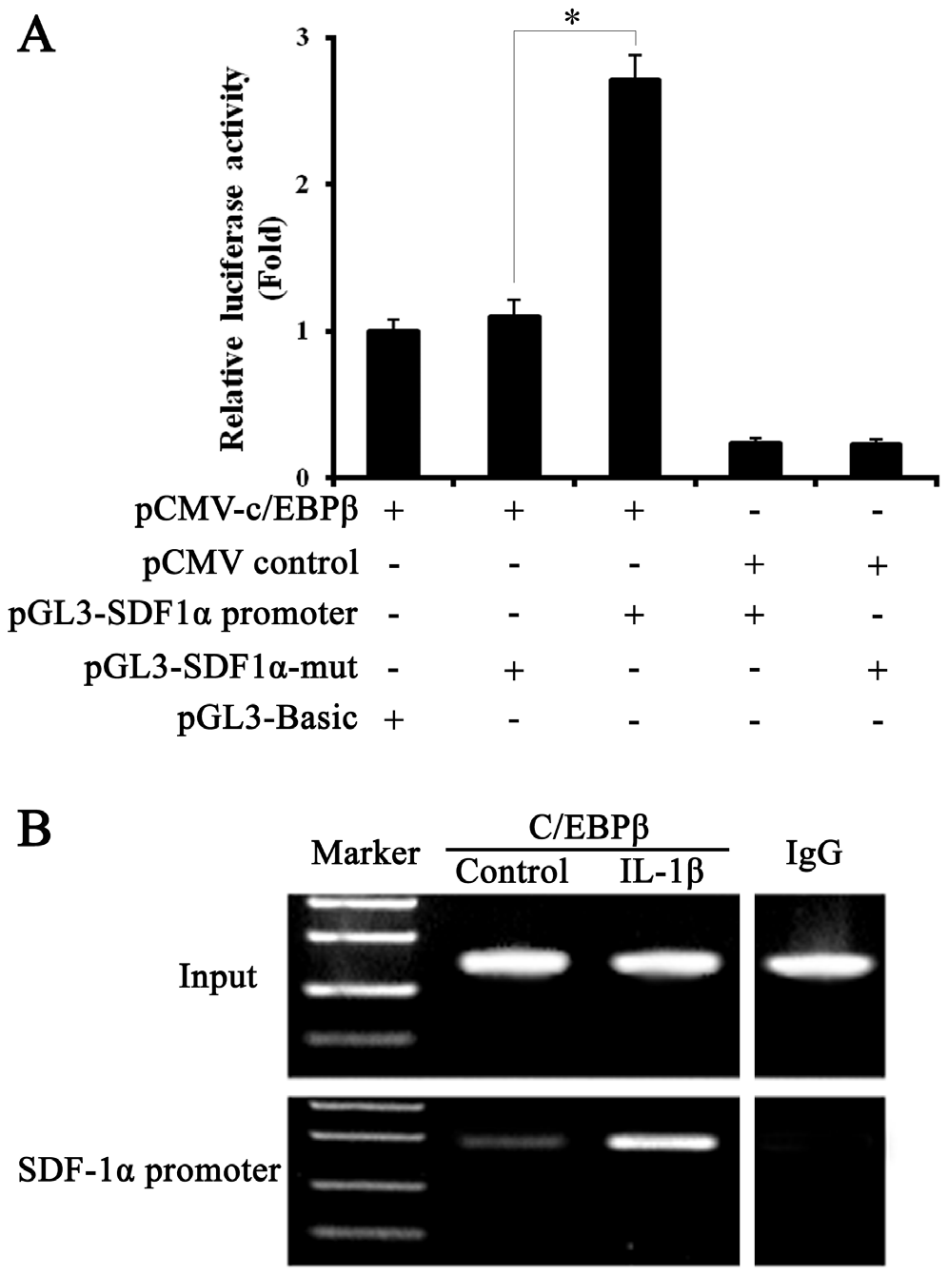
Analysis of C/EBPβ interaction with the SDF-1α promoter. **A,** luciferase assay, different plasmids were co-transfected into normal EPCs. After 48 h, luciferase activity was detected by dual specificity reporter gene kits (Promega). **B,** ChIP analysis of C/EBPβ binding to the SDF-1α promoter region in EPCs under 10 ng/ml IL-1β or control vehicle (DMSO) treatment for 24 hours. The input lane shows the starting chromatin extracts. All values are the means ± SD of three replicates, ^*^ *P*<0.05.

Luciferase assays showed that transfection with C/EBPβ and the SDF-1α promoter induced an increase of almost 3-fold in luciferase activity over C/EBPβ alone, while in the absence of C/EBPβ the activity of the SDF-1α promoter was reduced to approximately 20% (Fig. 3A). ChIP analysis of C/EBPβ binding to the SDF-1α promoter region in EPCs in the presence of 10 ng/mL IL-1β or vehicle control (Fig. 3B) confirmed that C/EBPβ binds to the SDF-1α promoter.

### Silencing of C/EBP-β reduces expression of SDF-1α

**Figure 4:**
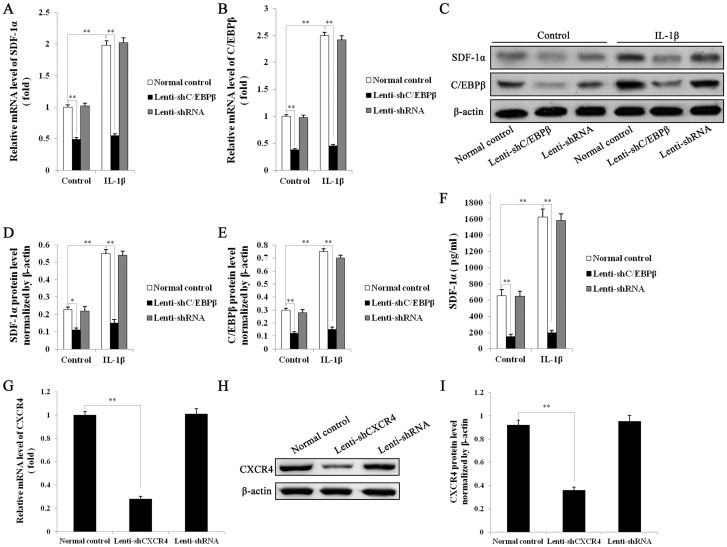
mRNA and protein expression of SDF1α and C/EBP-β by transfected EPCs, analyzed by real-time quantitative RT-PCR, western blotting and ELISA. Untreated EPCs or EPCs transfected for 12-shC/EBPβ or Lenti-shRNA, were treated with 10 ng/mL IL-1β or control vehicle (DMSO) for 24 hours, then mRNA levels of SDF1α (A) and C/EBP-β (B) were detected by Q RT-PCR. GAPDH served as control. C, protein levels of SDF1α and C/EBP-β were detected by western blot analysis. β-actin served as loading control. Quantitative analysis of SDF1α (D) and C/EBP-β (E) protein levels normalized to β-actin. F, Levels of secreted SDF1α in the medium of the above EPC cultures (Normal EPCs, Lenti-shC/EBPβ, Lenti-shRNA-EPCs) were determined by ELISA. mRNA and protein expression of CXCR4 by transfected RAW264.7 cells, analyzed by real-time quantitative RT-PCR and western blotting. **G,** Untreated RAW264.7 cells or RAW264.7 cells transfected with Lenti-shCXCR4 or Lenti-shRNA for 48 h, were subjected to Q RT-PCR to measure the mRNA levels of CXCR4. Results were normalized to GAPDH expression. **H,** Protein levels of CXCR4 were analyzed by Western blot. β-actin served as loading control. **I,** Quantitative analysis of CXCR4 protein level normalized to β-actin. All values are the means ± SD of three replicates, ^*^ *P*<0.05, ^**^ *P*<0.01.

Control EPCs or EPCs transfected with Lenti-shC/EBPβ or Lenti-shRNA in the presence or absence of 10 ng/mL IL-1β were used for QRT-PCR to assess mRNA expression of SDF-1α (Fig. 4A) and C/EBP-β (Fig. 4B), while protein levels of SDF-1α and C/EBP-β were detected by western blot analysis with β-actin as loading control (Fig. 4C). Protein levels were quantitated to analyze SDF-1α (Fig. 4D) and C/EBP-β (Fig. 4E) levels, normalized to β-actin. SDF-1α secretions in the supernatants of the above EPC cultures were determined by ELISA (Fig. 4F). All these results demonstrate that transfection of EPCs with lenti-shC/EBPβ reduced both mRNA and protein levels of SDF-1α and C/EBP-β, completely blocking the inductive effect of IL-1β.

### Silencing of CXCR4 in RAW264.7 cells

We hypothesized that secretion of SDF-1α by EPCs could recruit RAW264.7 cells through their expression of CXCR4. To test this, we knocked down CXCR4 expression in RAW264.7 cells. Untreated RAW264.7 cells or RAW264.7 cells transfected with Lenti-shCXCR4 or Lenti-shRNA for 48 h were analyzed by Q RT-PCR to detect CXCR4 mRNA levels (Fig. 4G). GAPDH served as control. Quantitative analysis of CXCR4 protein was performed by western blot (Fig. 4H and I); β-actin served as loading control. Transfection with Lenti-shCXCR4 effectively blocked mRNA expression of CXCR4, significantly reducing the protein level.

### C/EBPβ mediates the chemotactic response of RAW264.7 cells, which is inhibited by silencing CXCR4

**Figure 5:**
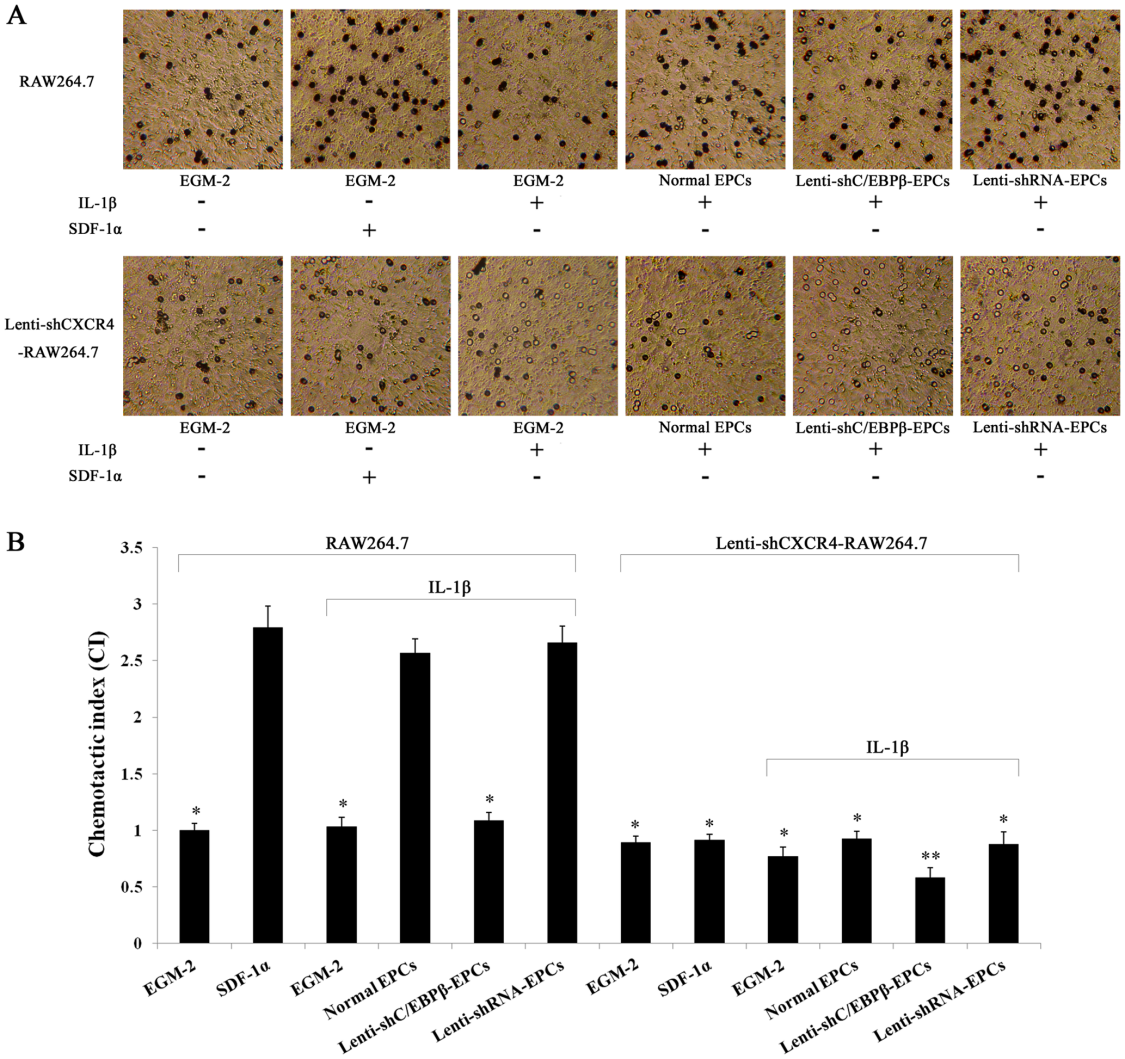
Chemotaxis of RAW264.7 cells. **A,** Cell migration was measured in chamber wells with the upper and lower chamber separated by an 8 µm pore size polycarbonate membrane. Upper chamber, untreated RAW264.7 or Lenti-shCXCR4-RAW264.7 cells; lower chamber, EGM-2 endothelial medium without or with IL-1β (10 ng/mL in EGM-2) or SDF-1α (100 ng/mL in EGM-2) or the supernatants of normal EPCs or of Lenti-shC/EBPβ-transfected or Lenti-shRNA-transfected EPC cultures treated with IL-1β (10 ng/mL) for 24 hours. **B,** Chemotactic index (CI) was calculated. All values are the means ± SD of three replicates, ^*^ *P*<0.05, ^**^ *P*<0.01 vs. normal EPCs.

We next investigated whether C/EBPβ and the SDF-1α receptor CXCR4 are involved in chemotaxis of RAW264.7 cells. Chemotaxis of RAW264.7 cells towards EPCs increased in the presence of IL-1β but this increase was blocked when the EPCs were transfected with Lenti-shC/EBPβ. The addition of IL-1β alone in the absence of EPCs had no effect on the migration of RAW264.7 cells (Fig. 5A, B). The increase in response to IL-1β was almost completely blocked when RAW264.7 cells were transfected with lenti-shCXCR4. Thus the receptor CXCR4 is largely responsible for mediating the activation of SDF-1 signaling in response to IL-1β, in agreement with our hypothesis, and this signaling pathway involves C/EBPβ. These results suggest that binding of SDF-1α to its receptor CXCR4 is important in recruiting RAW264.7 cells.

## Discussion

CXCR4 is constitutively highly expressed by circulating human monocytes and CD34+ hematopoietic precursors which can give rise to osteoclasts [25], [30], [31], while SDF-1 is also constitutively expressed by immature cells of the osteoblast lineage as well as vascular endothelial cells within bone [32], and is overexpressed in inflammatory conditions and tumors [33]. SDF-1 acts as a chemoattractant and is known to be critical for the homing of hematopoietic cells to the bone marrow and their subsequent localization and retention in appropriate niches [32] and it has been shown to stimulate chemotactic recruitment of circulating monocytes and promote their early differentiation and survival, thereby resulting in an increase in bone resorptive activity [18], [20], [34], [35]. Thus SDF-1 expressed by bone endothelial cells, marrow stromal cells and pre-osteoblasts may selectively target circulating osteoclast precursors, promoting their migration to appropriate perivascular sites for development and differentiation.

In addition to its effects on osteoclastic cells however, SDF-1 also acts as a chemoattractant for marrow stromal cells and pre-osteoblasts, and the accumulation of both pre-osteoclasts and pre-osteoblasts at sites of bone regeneration would likely result in increased bone turnover. For the repair of bone defects therefore treatment with SDF-1 alone will not improve bone formation. Nevertheless the increase in bone turnover induced by SDF-1 has a permissive effect on bone formation, enhancing the effects of other osteoinductive agents such as BMP-2 [36].

The transcription factor C/EBPβ is a member of the CCAAT/enhancer binding protein (C/EBP) family and is a major regulator of transcription of the SDF-1 gene in response to cytokines and cell confluence; however the regulation of this process is complex [25], [37], [38]. In leukemic cells, C/EBPβ was found to bind to the promoter region of SDF-1 and regulate its expression [39]. C/EBP family members are mainly expressed by cells of the macrophage and granulocytic lineage and are involved in regulating inflammation in a variety of cell types [40]. They are involved in regulating expression of genes restricted to these lineages, such as the M-CSF receptor [41], [42]. Of the 6 known isoforms of C/EBP, the β and δ isoforms have been shown to be upregulated by inflammatory stimuli [38], and their activity in turn is regulated by pro-inflammatory cytokines such as IL1-β, an effect which can be blocked by a specific inhibitor of mitogen-activated protein kinase (MAPK) [24]. IL-1β has previously been shown to up-regulate DNA binding activity by C/EBP family members in other cell types [43] and this activation was mediated, at least in part, by a MAP-kinase signaling pathway [24]. In addition, SDF-1 has been shown to be important in recruiting circulating osteoclast precursors, as well as stimulating osteoclast maturation [25].

Angiogenesis in bone is closely linked to bone resorption [44]. EPCs are known to both produce [12] and respond to SDF-1 [45]. We hypothesized that circulating IL-1β, an inflammatory cytokine, could act via C/EBPβ to regulate SDF-1 production by EPCs and thus recruit circulating osteoclast precursors to sites of angiogenesis within bone.

Our results showed that mouse bone marrow-derived EPCs respond to IL-1β by upregulating protein levels of both SDF-1α and C/EBPβ. A luciferase assay confirmed that C/EBPβ acts on the SDF-1α promoter in these cells, and that binding of C/EBPβ to the SDF-1 promoter is promoted by IL-1β. Silencing of C/EBPβ reduces both RNA and protein expression of SDF-1 and C/EBPβ, in the presence or absence of IL-1β.

We confirmed that silencing of CXCR4 in the osteoclastic precursor RAW cells resulted in reduced mRNA and protein expression of CXCR4. We then used these cells to investigate the response of RAW264.7 cells to migratory factors in EPC conditioned medium. Using a chemotaxis assay, we show that RAW264.7 cells migrate towards conditioned medium from EPCs treated with IL-1β. This chemotactic effect can be abolished by silencing C/EBPβ in EPCs, and is almost completely blocked by silencing CXCR4 in RAW264.7 cells.

The use of the RAW264.7 cell line is one limitation of this study, since these cells are an immortalized tumor line and may therefore act differently from normal primary osteoclasts. Nevertheless they are a well-established model of pre-osteoclasts and of osteoclast differentiation, exhibiting characteristics of pre-osteoclasts such as migration, differentiation in response to RANKL, and expression of MMPs and bone resorption by the mature osteoclastic cells [34], [46], [47], [48], [49] and demonstrated to show the same responses as primary bone marrow cells [50]. Consequently we believe they are a valid model for use in our study.

Although many of the steps in this process have already been demonstrated in other cell types, our findings elucidate the mechanism involved in endothelial cell signaling to osteoclasts. Our findings show that EPCs respond to an inflammatory stimulus such as IL-1β, which is likely to be released in the graft microenvironment, by upregulating SDF-1 expression. Further, they demonstrate that SDF-1 produced by EPCs binds to its receptor CXCR4 in RAW264.7 cells, and that C/EBPβ is necessary for the response to SDF-1. This is the first report to clearly demonstrate the requirement for C/EBPβ in the SDF-1 response and to show that osteoclast-like cells respond to SDF-1 by migrating towards the source of this cytokine. Our results connect the links in this chain to show how the tissue response to the invasive procedure of grafting releases inflammatory cytokines which stimulate incoming EPCs to recruit the host’s own osteoclast precursors. These then differentiate into mature osteoclasts which participate in graft resorption and bone remodeling, resulting in improved integration of the graft with the host bone. These findings improve our understanding of the graft remodeling process and may lead to better bone grafts which can integrate more quickly into host bone, speeding up the healing process.
